# Supplementary material for: Impact of Visceral Leishmaniasis on Local Organ Metabolism in Hamsters
Source: Metabolites. 2022 Aug 27;12(9):802. doi: 10.3390/metabo12090802 (PMC9506185; doi:10.3390/metabo12090802)
Supplement: Supplementary file 1 [file metabolites-12-00802-s001.zip › SI/Method_supplemmental_tables S6-S8.pdf]

Table S6. LC-MS instrumental methods

**Instrumental methods**			
Method Duration	7.50 min		
Chromatogram Peak Width	6 seconds		
Use Lock Masses	Off		
Exclusion list	144.98		
	235.21		
	285.01		
	311.08		
	314.13		
	371.10		
**LC parameters**			
**Time (min)**	**Flow (mL/min)**	**%B (Acetonitrile + 0.1% Formic Acid)**	**Curve**
0.00	0.500	2	5
1.00	0.500	2	5
2.50	0.500	98	5
4.50	0.500	98	5
5.50	0.500	2	5
7.50	0.500	2	5
7.50	Stop Run		
**Divert valve parameters**			
Switch at	0.2 min		
**MS parameters**			
Source			
Aux Gas Heater Temperature	350.0ºC		
Spray Voltage	3.80 (+) / 3.0 (-) kV		
Capillary Temperature	320ºC		
S-lens RF Level	50.0		
Sweep Gas Flow Rate	0		
Aux Gas Flow Rate	10		
Sheath Gas Flow Rate	35		
Full MS			
Scan Range	100 to 1500 *m/z*		
AGC Target	3E6		
Maximum IT	246 milliseconds		
Resolution	70,000		
dd-MS^2			
(N)CE/ Stepped	NCE: 20, 40, 60		
Resolution	17,500		
Maximum IT	54 milliseconds		
AGC Target	1E5		
Fixed First Mass	---		

Isolation Window	1.0 *m/z*
Top N	5
dd Settings	
Dynamic Exclusion	10.0 seconds
Charge Exclusion	---
Intensity Threshold	1.5E5
Exclude Isotope	On
Apex Trigger	---
Peptide Match	preferred
Min. AGC Target	8.00E3

Table S7. MZmine data analysis parameters.

Data analysis method (MZmine2 version 2.35)			
		Polarity	
		Negative	**Positive**
**MS^1**	Noise Level	4.0E5	4.0E5
**MS^2**	Noise Level	1.0E3	1.0E3
**Chromatogram Builder**	*m/z* Tolerance (ppm)	10.0	10.0
	Minimum Time Span (min)	0.01	0.01
	Minimum Height	1.2E6	1.2E6
	Mass List	masses	Centroid
**Chromatogram Deconvolution**	Algorithm	Local MINIMA	Local MINIMA
	RT Range for MS2 Scan Pairing (min)	0.1	0.1
	m/z Range for MS2 Scan Pairing (Da)	0.01	0.01
	Search minimum in RT range (min)	.08	.08
	Minimum absolute height	1.2E6	1.2E6
	Chromatographic threshold	20	20
	Min ratio of peak top/edge	1.19	1.19
	Minimum relative height	26	26
	Peak duration range (min)	0.01-1.00	0.01-1.00
**Deisotoping**	Representative Isotope	Lowest *m/z*	Lowest *m/z*
	m/z Tolerance (ppm)	10.0	10.0
	Maximum Charge	3	3
	Retention Time Tolerance (min)	0.1	0.1
	Monotonic Shape	Checked	Checked
**Alignment**	Retention Time Tolerance (min)	0.25	0.25
	m/z Tolerance (ppm)	10.0	10.0
	Weight for *m/z*	1	1
	Weight for Retention Time	1	1
**Row Filtering**	Remove previous peak list	Disabled	Disabled
	Retention Time (min)	0.20 – 7	0.20 – 7
	Minimum Peaks in a Row	3	3
	Keeps Only Peaks with MS^2 Scans	Enabled	Enabled

Table S8. GNPS parameters.

Precursor Ion Mass Tolerance	0.02 Da
Fragment Ion Mass Tolerance	0.02 Da
Min Pairs Cos	0.7
Minimum Matched Fragment Ion	4
Maximum shift between precursors	500 Da
Network TopK	10
Maximum connected component size (Beta)	100
Library Search Min Matched Peaks	4
Score Threshold	0.7
Search Analogs	Do Search
Maximum Analog Search Mass Difference	100 Da
Top results to report per query	1
Minimum Peak Intensity	0.0
Filter Precursor Window	Filter
Filter Library	Filter
Filter peaks in 50 Da window	Filter
Normalization Per file	Row Sum Normalization (Per File Sum to 1,000,00)
Aggregation Method For peak abundance per group	Mean
PCoA Distance Metric	Cosine
