# Supplementary figures and images for: Impact of Visceral Leishmaniasis on Local Organ Metabolism in Hamsters
Source: Metabolites. 2022 Aug 27;12(9):802. doi: 10.3390/metabo12090802 (PMC9506185; doi:10.3390/metabo12090802)

**A**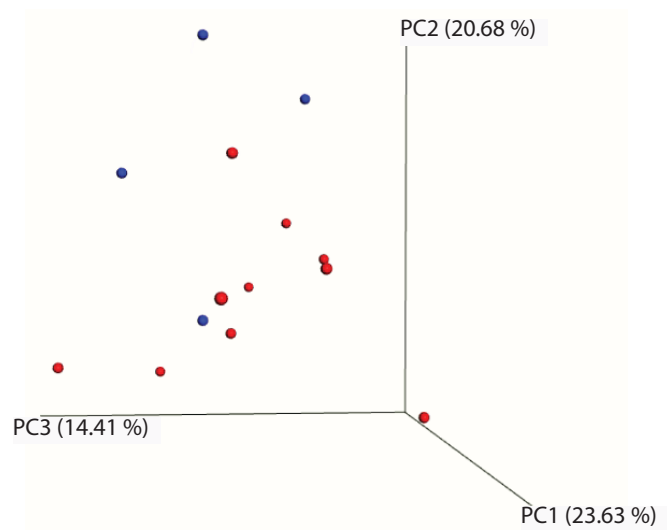**B**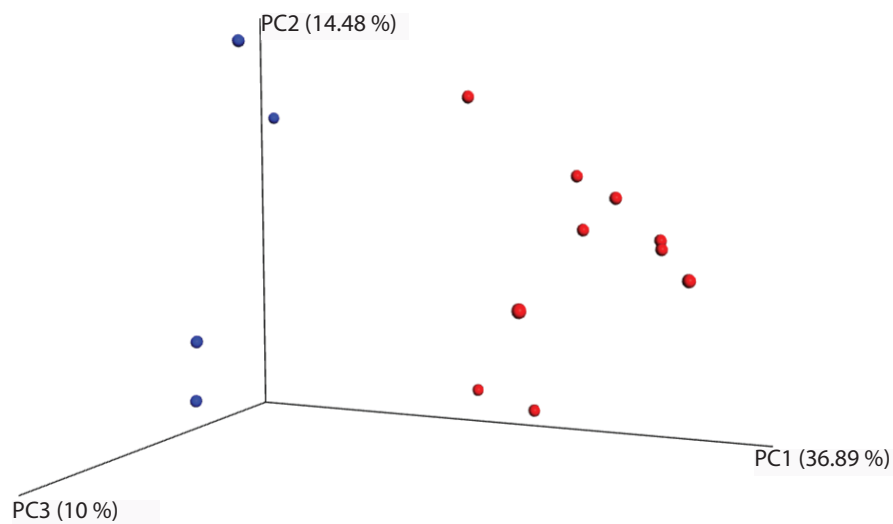**C**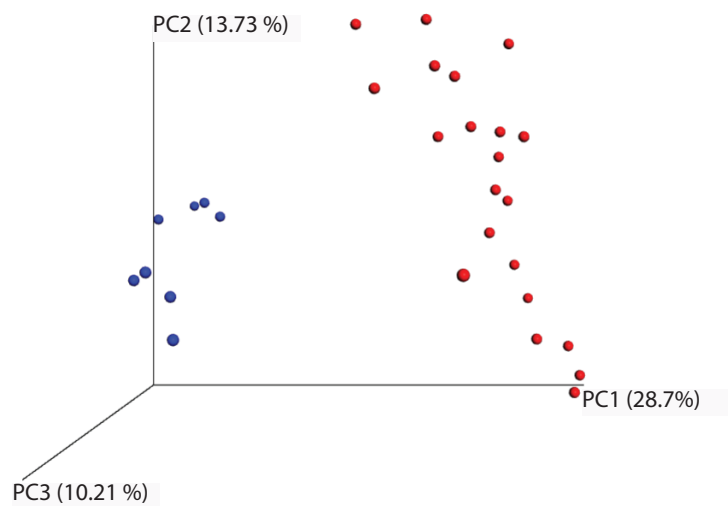**D**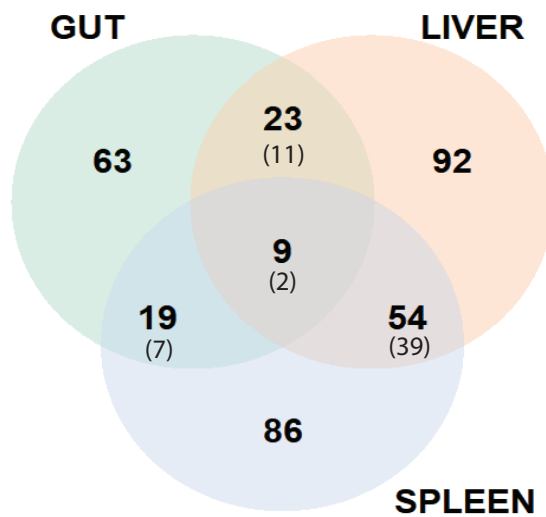

Supplement: Supplementary file 1 [file metabolites-12-00802-s001.zip › SI/FigS1_Leishmania_final.pdf]

**A**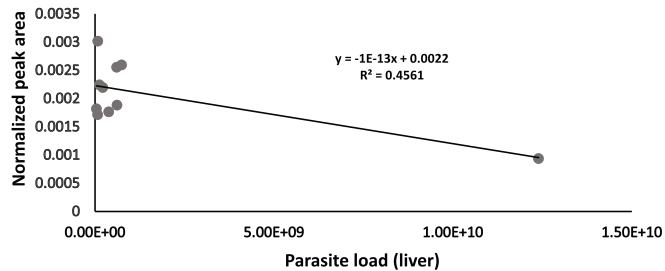**B**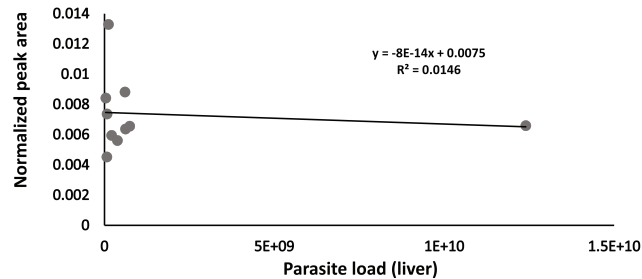**C**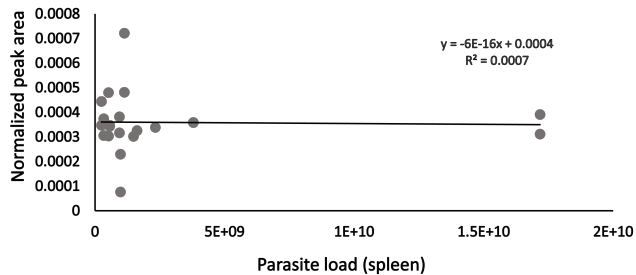**D**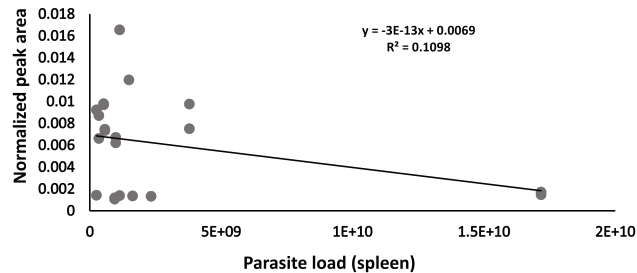

Supplement: Supplementary file 1 [file metabolites-12-00802-s001.zip › SI/FigS2_leishmania.pdf]

**A**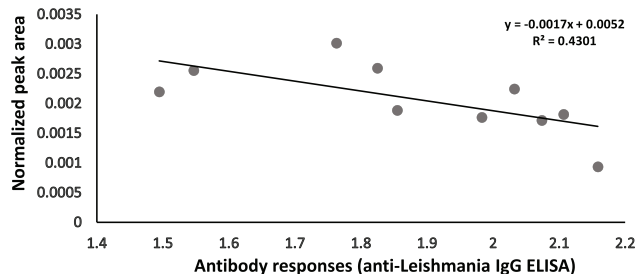**B**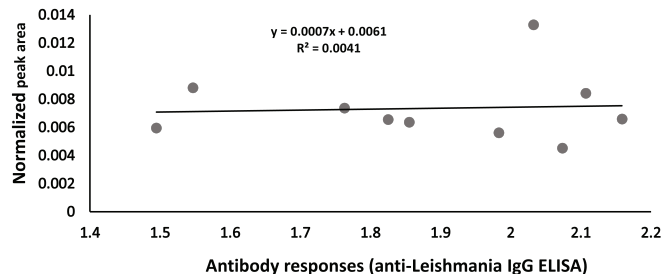**C**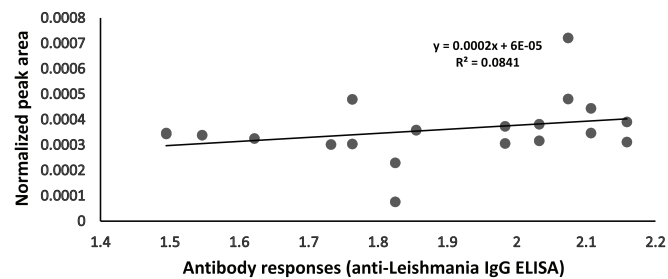**D**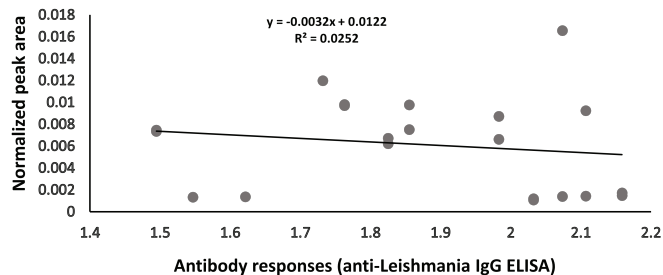**E**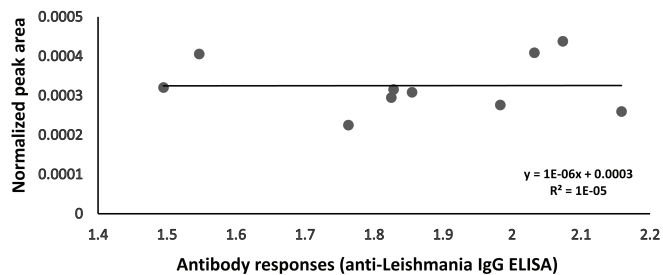**F**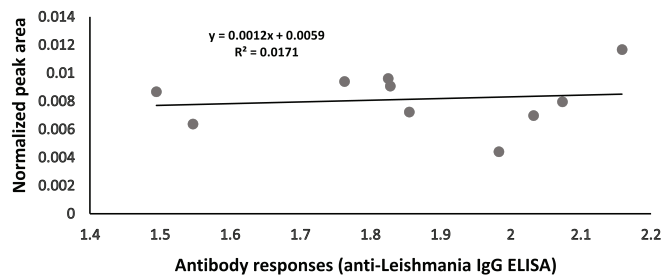

Supplement: Supplementary file 1 [file metabolites-12-00802-s001.zip › SI/FigS3_leishmania.pdf]
